# A Review of Current Computational Tools for Peptide–Protein Docking

**DOI:** 10.1002/jcc.70328

**Published:** 2026-02-13

**Authors:** Fábio G. Martins, Hélder A. Santos, Sérgio F. Sousa

**Affiliations:** ^1^ Department of Biomedicine, Faculty of Medicine LAQV/REQUIMTE, BioSIM, University of Porto Porto Portugal; ^2^ Department of Biomaterials and Biomedical Technology The Personalized Medicine Research Institute (PRECISION), University Medical Center Groningen, University of Groningen Groningen the Netherlands; ^3^ Departamento de Engenharia Química Faculdade de Ciências e Tecnologia, Universidade de Coimbra Coimbra Portugal

**Keywords:** drug discovery, molecular docking, molecular modeling, peptide therapeutics, peptides, peptide‐protein docking, protein‐ligand modeling, structure‐based drug design

## Abstract

Peptide–protein docking is an increasingly important technique in computational biochemistry and drug discovery, allowing researchers to predict interactions between peptides and proteins and guiding the development of peptide‐based therapeutics. This review provides a detailed overview of the current landscape of peptide–protein docking programs, emphasizing their importance and versatility. Through an extensive literature search, we identify and describe 14 dedicated peptide–protein docking programs, along with small‐molecule docking software that supports peptide docking. Additionally, we explore state‐of‐the‐art AI‐driven alternatives that are advancing the field. By describing the distinct features, methodological approaches, strengths, and inherent limitations of each docking tool, this review aims to support researchers in navigating the wide range of available docking programs and making well‐informed choices tailored to their specific research objectives.

## Graphical abstract

In this review, we compare 14 specialized peptide‐protein docking programs, adaptable small‐molecule docking tools, and emerging AI‐driven approaches. By clarifying how each method works, we offer researchers an accessible guide to selecting the most suitable tools for biochemical studies and peptide‐focused drug discovery.

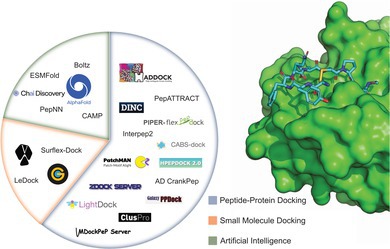

## 1 Introduction

Peptide–protein interactions are fundamental to numerous biological processes, including signal transduction, enzymatic regulation, and immune responses. Understanding how peptides bind to proteins provides valuable insights into their mechanisms of action, paving the way for the rational design of peptide‐based therapeutics [1, 2, 3]. Thus, peptide–protein docking has emerged as an important tool in computational biochemistry and drug discovery due to its ability to predict and analyze peptide–protein interactions [4].

Despite their therapeutic potential, peptide‐protein docking presents unique challenges. Peptides are highly flexible and can adopt multiple conformations. This means that, compared to the small molecules commonly studied, it is more challenging to predict peptide binding modes accurately [4, 5]. Additionally, peptide binding sites on proteins are often less well‐characterized than ligand binding sites, necessitating global searches across the protein surface. These factors demand advanced computational tools capable of handling the ample conformational space and dynamic nature of peptide‐protein interactions [4, 6].

This review will provide an in‐depth analysis of the molecular docking programs explicitly designed for peptide‐protein interactions. We will discuss their underlying methodologies, key features, and limitations and highlight the challenges and opportunities in applying these tools to peptide‐based therapeutic development. By comparing available docking software, this review aims to help researchers select the most appropriate tools for their specific peptide docking needs, ultimately advancing the design of peptide‐based therapeutics.

## 2 Background

Molecular docking is a computational tool, which has become essential in drug discovery. This method aims to predict the binding position of a molecule in relation to another. Using the known structures of the components, this method generates 3D conformations representing potential interactions within the complex [7]. Recent advances in computational power and algorithm development have significantly improved these methods [7, 8]. Initially, both the ligand and the protein remained in fixed conformations, in what is described as rigid docking. However, most programs now allow for ligand flexibility, allowing a much better exploration of the interaction between ligand and protein [8]. With the continuous advances in this field, protein flexibility is now also possible, with programs such as GOLD allowing for partial protein flexibility [9].

The initial phase of a docking study involves identifying the protein's binding site. If the binding site is already known, software often lets users focus the search on that specific area. If the binding site is unknown, blind docking can be performed to scan the entire protein surface for potential binding pockets [10]. Alternatively, specialized programs can predict the likely binding site, such as Fpocket [11] or CASTp [12].

A molecular docking program primarily consists of two key components: the search algorithm and the scoring function. The search algorithm explores various conformations and orientations of the ligand, and sometimes the receptor, to fit the ligand into the target receptor's binding pocket. The scoring function then evaluates these different poses, assigning scores to reflect the thermodynamics of the ligand–protein interaction. This scoring helps rank the poses to identify the most plausible binding models [10].

Different types of docking can be categorized based on the nature of the interacting molecules. Ligand‐protein docking is the most common type. It focuses on the interaction between small molecules (ligands) and larger macromolecules (proteins). These methods are usually very fast and efficient, making them useful for screening large libraries [13]. Protein–protein docking aims to predict how two proteins interact with each other, which is critical for understanding cellular processes and signaling pathways. However, the dynamic nature of protein interactions can complicate predictions. Rigid methods cannot accurately describe the adaptive interaction between proteins. Flexible methods, while more accurate, are computationally expensive, making them less practical for large‐scale studies [14]. Nucleic acid‐protein docking explores how proteins interact with DNA or RNA molecules. This is vital for understanding gene expression and regulation. Ligand‐nucleic acid docking focuses on the interaction between small molecules and DNA or RNA molecules. Despite nucleic acids being an important target for a variety of drugs, molecular docking software is usually developed and validated using protein‐ligand interaction as its focus [15]. However, new programs have been developed to try to model the interactions with nucleic acids accurately, such as PyDockDNA [16] and NLDock [17]. This work will focus on peptide‐protein docking. This type of molecular docking aims to study how peptides interact with proteins and has gained importance due to its relevance in understanding biological functions and designing peptide‐based therapeutics [18].

Peptides have gained prominence in medicinal chemistry due to their unique properties as therapeutic agents. These structures can interact with specific biological targets with high specificity and potency. They can act as hormones, neurotransmitters, or enzyme inhibitors [19]. Furthermore, peptides can act as agonists or antagonists, modulating receptor activity and playing a crucial role in regulating various cellular pathways [19, 20, 21]. This makes peptides very useful for treating a range of diseases, such as cancer or infectious diseases. Peptides have better selectivity for target sites and a lower likelihood of off‐target effects. Due to these advantages, the peptide drug market has been expanding rapidly. As a summary of the last 4 years, in 2021, 5 new peptide or peptide‐based drugs were approved by the FDA [22]; in 2022, 4 new peptides were approved [23]; in 2023, 5 more peptides were approved [24]; and in 2024, 2 more peptides were approved [25].

Beyond the steadily increasing number of approved peptide‐based drugs each year, a very large pipeline of candidates is currently in clinical development. Recent analysis reports that hundreds of peptide therapeutics, delivery systems, and peptide‐based vaccines are in various phases of clinical trials [26, 27, 28, 29]. This expanding clinical pipeline further reinforces the need for robust peptide–protein docking protocols to support design and optimization of new peptide drugs [26, 30].

Despite their therapeutic potential, peptide‐protein docking presents unique challenges compared to ligand‐protein docking. Peptides are much more flexible than small molecules and can adopt multiple conformations. This makes predicting how they will interact with proteins more complex and requires extensive computational sampling. Additionally, while ligand binding sites are often well‐characterized, peptide binding sites may not be known beforehand, requiring global searches across the entire protein structure for potential binding modes. The larger size and flexibility also complicate the identification of optimal binding sites and pose significant computational challenges for an accurate prediction. Consequently, fewer robust tools have been developed specifically for peptide‐protein docking compared to those available for the most common ligand‐protein interactions.

Molecular docking has emerged as a powerful computational technique to address some of the challenges of peptide design. By simulating the interaction between peptides and target proteins, docking methods allow researchers to predict the most likely binding conformations and affinities [31]. Docking tools adapted explicitly for peptide‐protein interactions consider the flexibility of peptides and their large conformational space, allowing for more accurate predictions compared to traditional small‐molecule docking methods [6]. As a result, molecular docking is crucial in accelerating the development of peptide‐based therapeutics.

## 3 Peptide/Protein Molecular Docking Software

**FIGURE 1:**
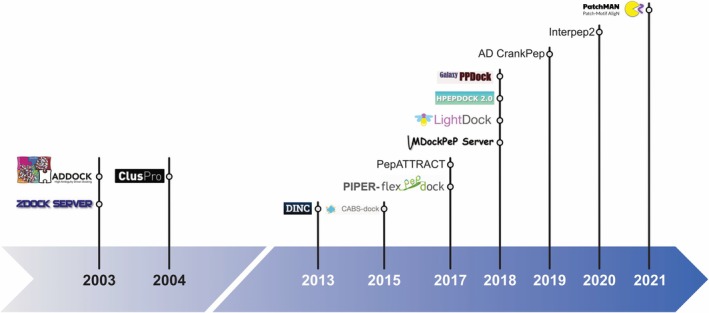
Chronological timeline of peptide–protein docking software releases.

This review presents a comprehensive overview of 14 distinct docking programs designed explicitly for peptide‐protein interactions. In Figure 1, the chronological timeline of the release of these peptide‐protein docking programs is presented.

While there are numerous alternatives available in the field of molecular docking, we have chosen to focus on these programs due to their prominence and the attention they have garnered in the scientific community. Some of the selected programs function as web‐based servers, allowing for easy access and use without extensive computational resources, while others require local installation, offering advanced features and customisation options for more experienced users. Table 1 summarizes the programs described in this review, including the program name, the release year, key references and websites.

**TABLE 1:** List of all peptide/protein docking software discussed in this article.

Software name	Release year	Latest update	References	Website
HADDOCK	2003	2024	[32, 33]	https://rascar.science.uu.nl/haddock2.4/
ZDOCK	2003	2014	[34, 35]	https://zdock.wenglab.org/
ClusPro	2004	2017	[36]	https://cluspro.org/
DINC	2013	2017	[37]	https://dinc.kavrakilab.org/
CABS‐Dock	2015	2019	[38, 39, 40]	https://biocomp.chem.uw.edu.pl/CABSdock
PepATTRACT	2017	2017	[41, 42]	https://bioserv.rpbs.univ‐paris‐diderot.fr/services/pepATTRACT/
PIPER‐FlexPepDock	2017	2017	[43]	http://piperfpd.furmanlab.cs.huji.ac.il/
GalaxyPepDock	2018	2018	[44]	https://seoklab.org/GalaxyPepDock/
HPEPDOCK	2018	2018	[6]	http://huanglab.phys.hust.edu.cn/hpepdock/
LightDock	2018	2023	[45, 46]	https://lightdock.org/
MDockPeP	2018	2022	[47, 48]	https://zougrouptoolkit.missouri.edu/mdockpep2/
AD CrankPep	2019	2021	[49]	https://ccsb.scripps.edu/adcp/
InterPep2	2020	2020	[50]	http://wallnerlab.org/InterPep2
PatchMAN	2021	2021	[51]	https://furmanlab.cs.huji.ac.il/patchman/

Table 2 presents key features of the peptide‐protein docking programs described in this review. It shows differences in docking mode, treatment of flexibility, requirements for prior knowledge of binding sites, accepted input types and software availability. The programs vary considerably in their capacity to account for peptide flexibility and receptor conformational changes, which is particularly important due to the inherent flexibility of peptides. Certain tools, such as HADDOCK and LightDock, enable fully flexible docking of peptide, at the expense of more computational power, whereas others, like ZDOCK and ClusPro, rely on rigid body approximations or allow only limited peptide flexibility. Requirements for binding site information also differ between the programs, with some necessitating a pre‐defined binding region, while others can perform global docking without prior knowledge. The information in the table assists in selecting the most suitable docking tool for a given peptide‐receptor system.

**TABLE 2:** Features of the peptide‐protein docking programs discussed in this work.

Software name	Docking mode	Prior site knowledge	Peptide flexibility	Receptor flexibility	Input type	Availability
HADDOCK	Local or global	Optional restraints	Fully flexible	Limited	Peptide Input Format	Server/Local
ZDOCK	Global	Not required	Rigid	Rigid	PDB	Both
ClusPro	Global	Optional	Rigid	Rigid	PDB	Server
DINC	Local	Required docking box	Fully flexible	Rigid or ensemble‐based	PDB or Fasta	Server
CABS‐Dock	Global	Not required	Coarse‐grained	Coarse‐grained	PDB	Server
PepATTRACT	Global	Not required	Coarse‐grained	Rigid	Fasta	Both
PIPER‐FlexPepDock	Global	Optional Constraints	Rigid	Rigid	Fasta	Server
GalaxyPepDock	Local	Template dependent	Semi‐flexible	Limited local flexibility	Fasta	Server
HPEPDOCK	Global or Local	Optional centre	Semi‐flexible	Rigid	Fasta	Local
LightDock	Global or Local	Optional restraints	Fully flexible	Fully flexible	PDB or Fasta	Server
MDockPeP	Global	Not required	Flexible refinement	Rigid	PDB	Both
AD CrankPep	Global or Local	Optional grid region	Fully flexible	Rigid	Fasta	Server
InterPep2	Global	Not required	Semi flexible	Rigid	PDBQT	Local
PatchMAN	Global	Not required	Rigid sampling. Flexible refinement	Rigid	Fasta	Local

### 3.1 Haddock

**FIGURE 2:**
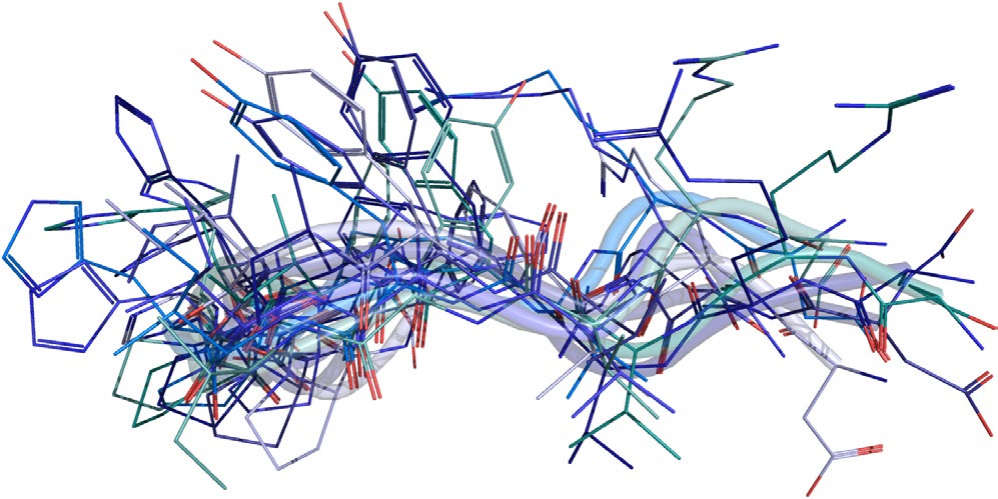
Superposition of multiple representative frames from a molecular dynamics trajectory of Angiotensin II (PDB: 6OS0 [52]). Backbone and side‐chain atoms from each frame are shown to visualize conformational heterogeneity and residue‐level flexibility.

HADDOCK (High Ambiguity Driven DOCKing) is a widely used docking software specializing in protein–protein, protein‐DNA, protein‐RNA, and protein‐peptide interactions. This program was developed in 2003 by the Bonvin lab at Utrecht University but has been consistently updated, with the last version released in 2024. HADDOCK distinguishes itself by not relying solely on geometric or energy‐based scoring to predict the interaction but by incorporating experimental data into the docking process. This hybrid approach combines data‐driven docking with traditional computational methods, providing higher accuracy [32]. Furthermore, a key feature of HADDOCK is its ability to account for flexibility during docking. This is particularly advantageous for peptide docking, as peptides exhibit greater flexibility than larger proteins. Consequently, one major challenge in peptide‐protein docking stems from the wide range of conformations that peptides can adopt (Figure 2). This structural fluidity expands the conformational space, complicating the identification of the bound state. Therefore, by accounting for flexibility, HADDOCK can provide more accurate predictions of peptide‐protein interactions, where structural adaptability plays a critical role [32].

HADDOCK employs a multistep, data‐driven docking, combining initial rigid‐body placement, semi‐flexible refinement and final explicit‐solvent optimization. The resulting docked structures are clustered based on backbone RMSD at the interface and ranked according to interaction energies and buried surface area [32].

The HADDOCK scoring function (HADDOCK score) consists of a linear combination of various energies and buried surface area. HADDOCK score differs for each docking stage, with different weights for each term. The first two stages are calculated using the following terms: van der Waals intermolecular energy, electrostatic intermolecular energy, desolvation energy, distance restraints energy, and buried surface area. For the last stage, the buried surface area is not used [32, 33].

HADDOCK is available both as a local program and as a web server. The web server, currently version 2.5 (https://wenmr.science.uu.nl/haddock2.4/), provides a user‐friendly interface for setting up and running docking simulations, making it accessible to researchers without extensive computational expertise. For more advanced users or those requiring greater customisation, a local version of HADDOCK 3 can be installed and run on their own computer [32, 33].

One of the biggest strengths of HADDOCK is its ability to incorporate experimental data, including NMR, mass spectrometry, and mutagenesis data. This ability to integrate multiple data sources often leads to more accurate predictions. The flexibility in the peptide chain is also a significant advantage of this program. However, while HADDOCK excels with data‐driven docking, it may struggle if no experimental data is available, as its core strength relies on restraints. Furthermore, peptide–protein interactions, especially with highly flexible peptides, can still be computationally challenging, and results may require further validation. Nevertheless, HADDOCK is a very useful program which has been used in multiple studies which focus on peptide–protein interactions. Recent studies which use HADDOCK include the use of antigenic peptides produced from *Streptococcus gallolyticus* in order to develop a proteomics‐based vaccine [53]. A different work used HADDOCK to develop a peptide‐based strategy against SARS‐CoV‐2 [54]. Another study modeled the interaction of different peptides with Human salivary alpha‐amylase with the aim of developing anti‐diabetic therapeutic agents [55].

### 3.2 ZDOCK

ZDOCK is a widely used protein–protein molecular docking program developed by the University of Massachusetts Medical School [35]. Developed in 2003, with the latest version released in 2014, this program uses a fast Fourier transform (FFT)‐based rigid‐body docking approach to predict the optimal binding mode between two molecules [34, 35]. After exploring the conformational space, ZDOCK uses its scoring function to evaluate the quality of each docked orientation.

Currently, two versions of ZDOCK can be used: version 2.3.2 and 3.0.2. In the case of version 2.3.2, the scoring function consists of Atomic Contact Energy (ACE) Statistical Potential, Shape Complementarity, and Electrostatics [35]. ACE is used to estimate desolvation [56]. The pairwise shape complementarity (PSC) contains favorable and penalty terms. The favorable term calculates the number of atom pairs between the receptor and the ligand within a specific distance cutoff. The penalty term prevents clashes by penalizing every core–core, surface‐core, and surface‐surface grid point overlap [56]. Lastly, the electrostatic energy is calculated using the Coulomb formula [56]. In the case of ZDOCK 3.0.2, the difference is that ACE is replaced by the improved Interface ACE (IFACE) [57]. Furthermore, M‐ZDOCK is also available. M‐ZDOCK is an adaptation of ZDOCK to predict the structures of cyclically symmetric multimers based on the structure of a protein [58].

ZDOCK is available as a standalone local program and as a web‐based server (https://zdock.wenglab.org/). The ZDOCK server has an easy‐to‐use interface for performing protein–protein and protein–peptide docking, making it accessible to researchers without extensive computational resources [34]. On the other hand, ZDOCK is also distributed as a local software package, which offers more flexibility for advanced users who need to integrate ZDOCK into larger workflows or handle sensitive data locally [35].

Although ZDOCK is traditionally applied to protein–protein docking, it has been demonstrated to perform well in peptide docking scenarios, primarily when the peptide structure is known or can be predicted with reasonable accuracy. However, considering that peptides adopt flexible and dynamic conformations, rigid‐body docking has limitations. To address this, users often employ a combination of ZDOCK and refinement techniques to accommodate peptide flexibility. Studies have shown that ZDOCK can produce reliable initial docking poses for protein–peptide complexes, which can be further refined using flexible docking algorithms or molecular dynamics (MD) simulation protocols. ZDOCK was used to study the interaction between Ebola virus VP35 and ubiquitin [59]. It was also useful for modeling the interaction of palmitoyl‐CoA with the PHD domain of Plant homeodomain finger protein 2 (PHF2) in a study about Palmitoylation‐driven PHF2 ubiquitination [60].

While ZDOCK offers significant advantages in terms of speed and accuracy for rigid‐body docking, its rigid nature can limit its application in cases where the peptide undergoes significant conformational changes upon binding. At the time of the publishing of the article, ZDOCK Server had completed over 6200 jobs from more than 1000 unique users. The server completed most docking jobs in an average of 11.5 min [34]. Future developments that integrate flexible docking options would enhance its performance in peptide docking scenarios. Version 3.0.2 [35] improved the protocol by improving the scoring function and prediction accuracy, but additional advancements will be necessary to address the flexibility challenge.

### 3.3 ClusPro

ClusPro is a widely used web server for protein–protein docking, which has also been effectively applied to protein‐peptide docking scenarios. Developed by researchers at Boston University and Stony Brook University and released in 2017, ClusPro employs a fast Fourier transform (FFT) based approach to perform global docking of proteins and peptides efficiently [36].

ClusPro generates candidate protein–protein complexes using a rigid‐body docking strategy, using PIPER, followed by clustering and refinement. Large numbers of possible complexes are sampled and scored based on physicochemical complementarity, and the most favorable solutions are grouped by structural similarity. Representative structures from the 30 most populated clusters are selected and refined [14, 36, 61].

For peptide–protein docking, the developers introduced PeptiDock, a specialized protocol for peptide docking that employs a motif‐based fragment search to improve accuracy [61].

Instead of a usual scoring function, ClusPro focuses on the centres of large low‐energy clusters. The program is based on the idea that the probability of a cluster is proportional to the number of structures it contains, suggesting that identifying the most populated clusters is more reliable than simply choosing the lowest‐energy structures. Using cluster centres as representative models, though not widespread, has proven effective in CAPRI (Critical Assessment of Predicted Interactions) experiments, supporting ClusPro's approach [36, 62]. Nevertheless, due to user requests, ClusPro now provides PIPER energy values for cluster centres and the lowest PIPER energy within each cluster. However, the developers claim that model ranking should be based on cluster population rather than energy scores. This is because these values do not account for entropic contributions, and the energy component weights are optimized for near‐native structures rather than for accurate thermodynamic modeling [36].

ClusPro is only available as a web server and is freely accessible for academic use (https://cluspro.org/). The server provides a simple interface where users can upload two protein structures in PDB format to perform the docking experiment [36].

One of the main advantages of ClusPro is its fully automated nature and user‐friendly web interface, making it accessible to researchers without extensive computational expertise. However, ClusPro also has some limitations. As a rigid‐body docking method, it may struggle with proteins that undergo significant conformational changes upon binding [36].

In conclusion, ClusPro represents a robust and accessible tool for protein–protein docking, offering a balance between computational efficiency and accuracy. Its success in various applications and benchmarks has established it as a valuable resource in the field of structural biology [63, 64, 65, 66]. ClusPro was used to study the interaction of gliadin peptides to HLA‐DQ, a cell surface receptor protein, and T‐cell receptors to identify polymorphic interaction sites that could be used for diagnosis or targeted drug development [63]. ClusPro was also used for the docking of a selection of peptides targeting GRP78‐dependent SARS‐CoV‐2 cell entry [64]. In a study on re‐epithelialization in chronic, non‐healing diabetic foot wounds, ClusPro was used to study the interaction of a novel peptide with keratinocyte growth factor, a major modulator of this process [65]. Another study used this program to identify epitopes on the Dengue virus envelope with potential for diagnosis [66]. While it excels in many scenarios, users should know its limitations, mainly when dealing with highly flexible proteins. As the field of protein docking continues to evolve, future improvements to ClusPro may address these challenges, further enhancing its utility in predicting peptide–protein interactions.

### 3.4 DINC

DINC (Docking INCrementally) is a molecular docking tool developed at Rice University to address the challenges of docking large ligands, particularly peptides. DINC 1.0 was originally released in 2013, with version 2.0 being released in 2017. Introduced initially as a meta‐docking strategy, DINC has evolved into a solution for predicting binding modes of protein‐peptide complexes. The tool was created to overcome the limitations of traditional docking methods, which often struggle with ligands with more than 10 flexible bonds [37].

DINC addresses the challenge of docking highly flexible ligands by using an incremental docking strategy. Instead of attempting to dock the entire ligand at once, this program progressively builds and docks the ligand in multiple stages, using AutoDock 4. This method allows an efficient exploration of conformational space and is able to handle ligands with many degrees of freedom [37].

The scoring function used by DINC is AutoDock's semi‐empirical free energy force field. This scoring method evaluates protein‐ligand interaction energy using pairwise evaluations with predefined weights, including parameters for dispersion/repulsion, hydrogen bonds, electrostatics, and desolvation [37].

DINC is available as a web server. The current version, DINC 2.0, provides a user‐friendly interface accessible at http://dinc.kavrakilab.org. The program requires that the peptide and the protein are in .pdb format and allows the user to use an automatically designed docking box or a user‐defined one [37].

The primary advantage of DINC has been its ability to be up to two orders of magnitude faster than AutoDock's standard protocol without sacrificing accuracy. This speed‐up makes it particularly useful for applications involving large‐scale virtual screening or exploring complex protein‐peptide interactions. However, like all computational methods, DINC has limitations. While efficient, its incremental approach may not capture the full range of possible binding modes for highly flexible ligands. It also requires the peptide to be in .pdb format, unlike other programs requiring only the sequence.

In 2025, DINC‐ensemble was released. This extension of DINC introduces receptor flexibility by docking peptides with an ensemble of receptor conformations, rather than a single rigid structure. This method generates and ranks binding modes across all receptor‐ligand pairs, returning both the best‐scoring ligand poses and a ranked list of receptor conformations that favor binding. DINC‐ensemble is available as a python package and a web‐server available in: https://dinc‐ensemble.kavrakilab.rice.edu/ [67].

DINC has been used in a work that aimed to identify HLA‐C03:02‐restricted CD8+ T‐cell epitopes from the HIV‐1 p17 matrix protein. DINC was used to perform molecular docking studies on various HLA‐C03:02‐restricted epitopes derived from HIV‐1 proteins, providing insights into the structural stability and binding dynamics of peptide‐HLA interactions. In another work, DINC was employed to predict receptor‐ligand interactions between antiviral peptides and the A36R protein of the monkeypox virus. The study identified three peptides with the best binding energies, demonstrating DINC's utility in screening potential therapeutic agents [68]. In the article developed by Jandl et al., DINC was used to investigate the interactions between peptide‐fluorophore conjugates and cancer DNA for amplification‐free detection methods. The study focused on designing a hydrogel‐based assay that enhances signal detection of mutations in the EGFR oncogene, demonstrating DINC's capability to help study binding interactions [69]. In conclusion, DINC's innovative incremental approach makes it a valuable resource for researchers studying protein‐peptide interactions.

### 3.5 CABS‐Dock

CABS‐Dock is a computational method for protein‐peptide molecular docking that stands out for its ability to handle significant conformational flexibility of both the peptide and the protein receptor. Originally released as a web server in 2015, a standalone version was released in 2019. Developed as an extension of the CABS (C‐Alpha, Beta, and Side‐chain) coarse‐grained protein model, CABS‐Dock is a powerful tool for predicting protein‐peptide interactions without requiring prior knowledge of the binding site [39, 70].

This program performs peptide‐protein docking using a coarse‐grained representation that allows full peptide flexibility and limited receptor flexibility during sampling. The method generates multiple peptide binding poses on the protein surface, which are evaluated using a knowledge‐based statistical potential and clustered to identify the most probable binding modes [71]. Selected models are then reconstructed to all‐atom resolution and refined using Modeler [72], yielding a small set of representative peptide‐protein complexes [38, 70, 73].

As stated above, CABS‐Dock is available as a standalone program and web server (https://biocomp.chem.uw.edu.pl/CABSdock). The web server provides a user‐friendly interface that provides easy access to the basic functionalities of CABS‐Dock. It allows for quick setup and execution of docking simulations through a simple web interface [38]. On the other hand, the standalone version of CABS‐Dock is a more robust and customisable tool that is implemented as a Python package. This standalone version offers advanced features and support for large‐sized systems and provides users with complete control over the docking simulation from initial setup to results analysis. The standalone package is particularly useful for integrating CABS‐Dock into existing computational pipelines or for users who require more control over the docking process [40].

CABS‐Dock offers several advantages. Its primary strength lies in its ability to simulate significant backbone flexibility of the entire protein‐peptide system in a reasonable computational time. This feature is particularly useful for modeling large‐scale conformational changes. Furthermore, CABS‐Dock can perform blind docking, making it valuable for exploring potential peptide binding locations. Moreover, the fact that CABS‐Dock is available both as a user‐friendly web server and as a standalone package offers flexibility for different user needs. However, using a coarse‐grained model while enabling efficient simulations may sacrifice some atomic‐level details. This could impact the accuracy of predictions for interactions that heavily depend on specific side‐chain orientations.

CABS‐Dock has been used in a study focusing on the interaction of antimicrobial peptides with multiple proteins from ESKAPE pathogens [74]. Other work used this program to study the interaction of nanobody‐derived peptides, which targeted the active‐state β2‐adrenergic receptor conformation [75]. Zhu et al. used CABS‐Dock to study the interaction of a specific peptide with the 20S proteasome core particle in order to better understand and control the opening of the α‐ring gate, which could be useful for the treatment of cancer and neurodegenerative diseases [76]. In a study focused on diagnosing cutaneous leishmaniasis, CABS‐Dock was used to dock novel synthesized peptides to detect specific proteins secreted by the *Leishmania* parasite during infection [77]. In conclusion, CABS‐Dock offers a balance between computational efficiency and conformational flexibility. Its ability to handle flexible docking without prior binding site information makes it a valuable tool for researchers studying protein‐peptide interactions.

### 3.6 PepATTRACT

PepATTRACT is a peptide‐protein docking program designed for blind, large‐scale docking experiments. Developed in 2015 (the current version was released in 2017), PepATTRACT addresses the computational cost of proteome‐wide peptide‐protein docking by enabling fully blind docking without prior knowledge of the binding site [41, 42].

As with most programs mentioned in this review, the PepATTRACT web server operates through a multi‐stage process. This program performs peptide‐protein docking using a coarse‐grained representation and a global rigid‐body search strategy. First, it generated idealized peptide conformations, docks them against the receptor and optimized their relative orientations using energy minimization. Docking solutions are scored with the ATTRACT force field, clustered based on structural similarity, and representative low energy models are selected as final predictions [42, 78, 79, 80].

The ATTRACT scoring function captures steric, electrostatic, and residue‐level interaction preferences and is designed to identify plausible binding modes rather than to estimate binding affinities. The server outputs a ranked set of the 50 lowest‐energy models, each representing the best structure from a docking cluster [41].

PepATTRACT was initially available as a standalone program and web server. The full pepATTRACT protocol, which includes flexible refinement using iATTRACT and molecular dynamics refinement and re‐scoring, is meant to be run locally and available through the ATTRACT web interface at http://www.attract.ph.tum.de/peptide.html. However, as of the date of writing, the web interface is not online. The simplified version of pepATTRACT, described above, is available as a web server (https://mobyle.rpbs.univ‐paris‐diderot.fr/cgi‐bin/portal.py#forms::pepATTRACT).

One of the primary advantages of PepATTRACT is its ability to perform fully blind docking without requiring binding site information. The web server version's speed (each run takes around 10 min) makes it particularly well‐suited for large‐scale *in silico* experiments [41]. However, the web server version, which only performs the rigid‐body stage, is a limitation compared to other programs described in this review.

PepATTRACT was used to dock a peptide‐based vaccine with Toll‐Like Receptor 4 in a study focusing on a potential new vaccine for chickens against *Toxoplasma gondii* [81]. Another work concerning the development of a novel peptide that targets tumor‐associated fibronectin and tenascin isoforms used this program to analyze the interaction between the new peptide and the target proteins [82]. PepATTRACT was also used to predict the affinity of a new peptide antagonist against periostin to overcome doxorubicin resistance in breast cancer [83]. In a work developed by Eggink et al., PepATTRACT was one of the programs used for the docking of a peptide mimetic of *N*‐acetylgalactosamine (GalNAc) to GalNAc‐specific lectins in search of a peptide capable of activating an immune response against ovarian cancer [84]. This program was used; PepATTRACT offers a fast, fully blind docking protocol suitable for large‐scale studies. While it has limitations, it provides a valuable tool for researchers.

### 3.7 PIPER‐FlexPepDock

PIPER‐FlexPepDock is a fragment‐based protocol designed for high‐resolution global peptide‐protein docking. Developed by researchers at the Hebrew University of Jerusalem and released in 2017, this program addresses the challenges of modeling highly flexible peptides on large receptor surfaces. It has shown remarkable accuracy and efficiency in predicting peptide‐protein interactions [43].

The protocol works through a divide‐and‐conquer approach, integrating three main components: the Rosetta fragment picker [85] for generating accurate peptide fragment ensembles, the already mentioned PIPER docking algorithm [86] for exhaustive fragment‐receptor rigid‐body docking, and Rosetta FlexPepDock [87] for flexible full‐atom refinement of the docked models. Short peptide fragments derived from the input sequence are first docked exhaustively onto the receptor, after which the most promising models are refined to full atomistic detail. Final predictions are obtained by clustering refined models and selecting representative structures from the top‐ranked clusters [43].

Model ranking relies on Rosetta‐based energy evaluation focused on the peptide–protein interface. Alternative scoring schemes were explored to improve binding specificity, with composite scores that emphasize interface and peptide contributions providing the most reliable discrimination of near‐native binding modes [88].

This program is available only as a web server (http://piperfpd.furmanlab.cs.huji.ac.il) and is accessible to the academic community. Users can submit jobs by providing the receptor structure and peptide sequence through a user‐friendly interface. The results include downloadable models and an interactive viewer for inspecting the top‐ranking predictions [43].

PIPER‐FlexPepDock offers several advantages. It demonstrates excellent performance, with a success rate of 52% in generating near‐native models (within 2.0 Å RMSD) among the top 10 ranked clusters [43]. The method is also efficient, thanks to its fast rigid‐body docking followed by targeted refinement. Additionally, it can handle cases where binding sites are known and cases where they are not. However, potential disadvantages might include computational cost for the refinement stage and the need for accurate initial fragment generation. Furthermore, the receptor cannot have more than 500 residues, and the peptide size must be between 4 and 15.

PIPER‐FlexPepDock has already been used in multiple studies. For example, Li et al. used this program to dock newly designed peptide inhibitors of Cyclophilin D as a potential treatment for acute pancreatitis [89]. Other work used PIPER‐FlexPepDock to study peptides that target and try to inhibit the RNA‐dependent RNA polymerase complex of SARS‐CoV‐2 [90]. This program was also used to study the APP9mer‐SD1 complex in order to provide a theoretical solution for how to determine a biomolecular structure with a highly flexible, disordered fragment [91]. PIPER‐FlexPepDock's integration of fragment‐based modeling, fast rigid‐body docking, and flexible refinement allows for efficient and accurate prediction of peptide–protein interactions.

### 3.8 GalaxyPepDock

GalaxyPepDock is a web‐based protein‐peptide docking tool that combines a similarity‐based approach with energy optimisation. Developed by Lee et al. and released in 2018, it has become a valuable resource for researchers studying protein‐peptide interactions [44].

GalaxyPepDock is a template‐based peptide‐protein docking method that identifies suitable complex templates from the PepBind database [92]. Candidate models are constructed using the GalaxyTBM tool [93] and refined with GalaxyRefine [94]. This hybrid strategy, combining template information with structural refinement, enables accurate prediction of peptide‐protein interactions [44].

The scoring function of GalaxyPepDock is not explicitly detailed. The server selects the top 10 models based on energy calculations, suggesting that the scoring function incorporates energy terms to evaluate and rank the generated models [44].

This tool is a user‐friendly web server that is accessible at https://galaxy.seoklab.org/cgi‐bin/submit.cgi?type=PEPDOCK. To use GalaxyPepDock, users must input a protein structure (in PDB format) and a peptide sequence [44].

GalaxyPepDock's similarity‐based approach allows for highly accurate predictions when similar complexes exist in the database. The user‐friendly server requires input from only the protein structure and peptide sequence. Additionally, GalaxyPepDock provides estimated accuracy for its predictions, allowing users to assess the reliability of the results. However, the method also has some limitations. Its performance may be reduced when no similar complexes are found in the database. The server also has size limitations, restricting proteins to 900 amino acids and peptides to 30 amino acids [44].

To study how human cytomegalovirus (HCMV) exploits host retrotransposons for enhanced viral fitness, GalaxyPepDock was used to study the interaction between ORF2p, a protein with endonuclease and reverse transcriptase domains, and the DNA polymerase subunit from HCMV. For that, they only used the candidate interaction site of ORF2p, which they called the ORF2p peptide [95]. Honrubia et al. used this program to dock a peptide, based on the 15 amino acid carboxyterminal sequence of human SARS‐CoV, to Syntenin‐1 as the first step in a computational protocol aimed at elucidating the functional relevance of SARS‐CoV proteins 3a and E in virus replication and virulence [96]. In the development of a novel peptide for the treatment of leukemias and lymphomas, GalaxyPepDock was used to dock the promising peptide with a protein that promotes survival in malignant cells [97]. Another example of the usefulness of this program was a study that designed a peptide‐based vaccine against the Omicron variant of SARS‐CoV‐2 and used this software for docking the peptide with Toll‐like Receptor 5 [98].

In conclusion, GalaxyPepDock offers a powerful and accurate method for predicting protein‐peptide interactions by combining database searching with energy‐based optimisation. While it performs exceptionally well when similar structures are available, future improvements could focus on enhancing its performance for novel interactions. Nevertheless, GalaxyPepDock is a significant advancement in protein‐peptide docking.

### 3.9 HPEPDOCK

HPEPDOCK is a specialized computational tool released in 2018 and developed by Huazhong University of Science and Technology researchers for docking peptides to protein targets [6].

HPEPDOCK performs peptide‐protein docking by first generating multiple peptide conformations to account for their inherent flexibility with MODPEP [99]. Then, the generated conformations are docked into the protein using a modified version of MDock [100]. The resulting models are evaluated with a knowledge‐based scoring function, ITScore‐PP, and optimized to identify energetically favorable binding poses. This approach allows efficient sampling of peptide binding modes, while providing a ranked set of representative peptide‐protein complexes [101].

HPEPDOCK 2.0 is available only as a web server, accessible at http://huanglab.phys.hust.edu.cn/hpepdock/. A Linux cluster supports the server and uses the SLURM Workload Manager for job scheduling. It does not require registration and can be freely accessed by users. It supports inputs in PDB and FASTA formats [6].

HPEPDOCK offers several advantages, including its ability to model flexible peptide conformations and its user‐friendly interface, which makes it accessible to researchers with varying levels of computational expertise. Because this program models flexibility through an ensemble of peptide conformations instead of running lengthy simulations, the server is very efficient, taking an average of 29.8 min for a global peptide docking and 14.2 for a local docking job [6]. These timings were obtained with benchmark sets of 62 protein‐peptide dockings for global docking and 57 for local docking, with an average peptide length of 9 amino acids [6]. However, the program does have some limitations, such as the potential need for significant computational resources, especially for extensive or complex docking studies.

HPEPDOCK is an increasingly popular program, with multiple studies using it for docking small peptides to large proteins [102, 103, 104, 105, 106]. Wang et al. used this program in the early steps of a multi‐phase computational protocol for the identification of novel antioxidant peptides obtained from soybean protein [102]. In another study, HPEPDOCK was utilized to investigate the interaction between peptides derived from *Osmanthus fragrans* during in vitro digestion and α‐glucosidase to explore their potential as inhibitors of this enzyme [103]. This program was also used to discover LL13, a new peptide with anti‐proliferative activity [104]. The multi‐step process ensures a detailed, accurate, and relatively fast prediction of peptide‐protein interactions, which is crucial for drug discovery and protein engineering applications.

### 3.10 LightDock

LightDock is a versatile and powerful protein–protein, protein–peptide, and protein–DNA docking protocol. Developed by Brian Jiménez‐García and colleagues, it is based on the Glowworm Swarm Optimization (GSO) algorithm [45]. It was originally released in 2017, with the latest version released in 2023 [46].

LightDock is a flexible peptide‐protein docking framework that uses a swarm‐based optimization strategy to explore ligand binding poses. The method can account for flexibility in both the receptor and the ligand and allows incorporation of additional information, such as residue restraints, to guide docking [45]. Models are evaluated using one of multiple knowledge‐based scoring functions, with the default being DFIRE, and representative low‐energy complexes are returned as predictions [107].

LightDock is available both as a standalone tool and as a web server. The standalone version, implemented in Python, allows for greater customisation and is suitable for users with programming experience who want to modify the protocol or run it on their computational resources. Furthermore, LightDock‐Rust is an implementation of the LightDock software in the Rust programming language, allowing a much faster execution of the program, with the only downside being the fact that only the DFIRE scoring function is available. On the other hand, the LightDock Server (https://server.lightdock.org/) is a user‐friendly web interface that provides easy access to the docking protocol without requiring installation or extensive computational resources. It is also written in the Rust programming language, allowing for faster speed. To use the server, the user must use .pdb files of the protein and peptide as input [45, 46].

This program offers several advantages, including its flexibility in accommodating different types of molecular interactions, the ability to incorporate experimental data as restraints, and its customisable nature. Using swarm intelligence allows for efficient exploration of the interaction energy landscape. Additionally, the availability of both standalone and server versions caters to different user needs. However, LightDock also has some limitations. Like many docking programs, its accuracy can be affected by the quality of input structures and the chosen scoring function. The computational cost can be high, especially for large systems or when using more complex scoring functions. Furthermore, while the flexibility options are beneficial, they may also increase the complexity of the search space and potentially lead to false positives.

In a study which focused on designing a multi‐epitope peptide vaccine against porcine rotavirus (PoRV), LightDock was used to model the docking of the vaccine construct with toll‐like receptors (TLR3 and TLR4), identifying stable interactions crucial for immunogenicity validation [108]. Another study, which aimed to create and evaluate a potential vaccine candidate against leishmaniasis, used LightDock to assess the binding interactions between a designed multi‐epitope vaccine candidate (MEVC) and the human TLR‐4 receptor [109]. Roque‐Borda et al., in an effort to develop protective alginate‐based microparticles for the antimicrobial peptide Ctx(Ile21)‐Ha, enhancing its stability and activity against intestinal infections, used LightDock to identify the interactions between Ctx(Ile21)‐Ha and bacterial receptors, which helped predict and optimize the peptide's binding affinity to pathogenic targets [110].

In conclusion, LightDock offers a novel, flexible, customisable approach to predicting protein–protein, protein‐peptide, and protein‐DNA interactions. Its implementation as both a standalone tool and a web server makes it accessible to a wide range of researchers.

While it has limitations, like all docking programs, its unique features and ongoing development make it a valuable tool for studying molecular interactions and guiding experimental research in structural biology and drug discovery.

### 3.11 MDockPeP

MDockPep (released in 2018) and its successor MDockPep2 (released in 2022) are ab initio protein‐peptide docking servers developed by researchers at the University of Missouri. These programs start with the peptide sequence and globally dock the flexible peptide to the receptor protein without previous knowledge about the binding site [47, 48].

MDockPep and MDockPeP2 perform peptide‐protein docking by first generating multiple peptide conformations using MODELER [72]. The generated peptide conformations are then docked onto the target protein using a hybrid strategy that combines global sampling with local flexible refinement using ZDOCK 3.0 [35] and Autodock Vina [111]. Candidate models are scored using a combination of physics‐based metrics from Autodock Vina, and an interface similarity metric which is calculated using PCalign [112]. The representative low energy complexes are selected as the final predictions [48].

Both MDockPep and MDockPep2 are available as web servers, making them easily accessible to researchers without the need for local installation. MDockPep can be accessed at https://zougrouptoolkit.missouri.edu/mdockpep, while MDockPep2 is available at https://zougrouptoolkit.missouri.edu/mdockpep2/download.html.

As with HADDOCK and HPEPDOCK, one of the main advantages of MDockPep and MDockPep 2 is its ability to account for the flexibility of peptides. Furthermore, it is possible to perform global docking without prior knowledge about the binding site. It also has the advantage of working with only the peptide sequence and not needing a 3D structure.

MDockPeP represents a significant advancement in protein‐peptide docking, which is a balance between computational efficiency and modeling accuracy used in multiple studies [113, 114, 115, 116]. This program was used in the development of a potential novel peptide‐based vaccine to predict the peptide–MHC interactions [113]. MDockPep was also used to predict the interaction of novel, designed peptides with the *S. salar* TLR5 ectodomain to enhance the immune response in salmon against bacterial infections [114]. In another work, this program was used to perform a blind docking study of a newly developed peptide and aerolysin, a virulence protein produced by the bacterial pathogen *Aeromonas sobria* [115]. Boldin et al. used MDockPep to study a novel peptide inhibitor of β‐Secretase‐1, which is linked to several amyloid‐linked neurodegenerative diseases [116]. Its ability to perform blind docking with peptide flexibility makes it particularly useful for cases where binding site information is unavailable or for large‐scale studies.

### 3.12 AD CrankPep

AutoDock CrankPep (ADCP) is an innovative approach to protein‐peptide docking that combines peptide folding and docking into a single process. Developed by Zhang and Sanner and released in 2019, ADCP has shown superior performance compared to other leading peptide docking tools, particularly for peptides up to 20 amino acids in length, making it a valuable tool for predicting protein‐peptide interactions and aiding in the development of peptide‐based therapeutics [49].

ADCP utilizes a modified version of the CRANKITE algorithm, originally designed for protein folding simulations [117]. ADCP samples a wide range of peptide conformations while keeping the receptor rigid [49, 118]. Candidate peptide poses are evaluated using a hybrid scoring function that considers both peptide conformational preferences and interactions with the receptor. This approach enables efficient identification of energetically favorable peptide–protein complexes [49].

ADCP is only available as a program that users can download and run on their local machines. It is distributed as part of the AutoDock suite of tools, which includes other popular molecular docking software like AutoDock Vina. ADCP can be installed on various operating systems, including Linux, macOS, and Windows, allowing researchers to perform peptide–protein docking calculations on their computational resources [49].

ADCP offers several advantages over traditional peptide docking methods. Its ability to simultaneously fold and dock peptides allows for more efficient and accurate predictions, especially for longer peptides. ADCP has demonstrated superior performance on benchmark datasets like LEADS‐PEP and peptiDB [49]. Additionally, ADCP is computationally efficient, with recent optimisations significantly reducing runtime. However, ADCP also has some limitations. It assumes a rigid receptor, which may not accurately represent the dynamics of protein‐peptide binding in all cases. The method may also struggle with highly constrained peptides or those with unusual structural features that are not well represented in its sampling approach.

AutoDock CrankPep offers a unique approach that combines folding and docking. It has been used by Tateto et al. to further elucidate the interaction between specific proline‐rich motifs of son of sevenless homologue 1 (SOS1) and the two Src homology three domains of the growth factor receptor‐bound protein 2 (GRB2) to better understand cell signaling pathways [119]. This program was also used in a similar study which focused on the Drk protein, the GRB2 homologue in *Drosophila* [120]. ADCP was also useful in the virtual screening stage of a study which aimed to identify antimicrobial peptides with inhibitory activity against the multidrug efflux pump of the *Pseudomonas aeruginosa* [121]. Another study used ADCP to dock oligopeptide substrates derived from SARS‐CoV‐2 polyproteins to the papain‐like protease (PLpro). This approach revealed the importance of the P2 proline in efficient PLpro catalysis, which was subsequently validated through experimental methods [122].

Autodock CrankPep's performance on benchmark datasets and ability to handle longer peptides make it a valuable tool for researchers studying protein‐peptide interactions and developing peptide‐based therapeutics. While it has some limitations, ADCP's innovative approach and demonstrated effectiveness make it a widely used method.

### 3.13 InterPep2

InterPep2 is a peptide‐protein molecular docking tool developed at the Division of Bioinformatics of Linköping University. Released in 2019 and updated in 2020, InterPep2 is a freely available tool that has shown significant performance enhancements over its predecessor, InterPep, and has been designed to provide more accurate and reliable predictions for peptide‐protein interactions [50].

InterPep2 predicts peptide‐protein interactions by generating multiple peptide conformations and matching them to structural templates using TM(template modeling)‐align [123], the Rosetta fragment picker [85] and fixbb [124]. Each peptide is aligned to the corresponding side of the template interface using the InterComp method [125]. Then, the templates are evaluated using a random forest regressor, trained to predict the DockQ‐score [126] for a given template based on sequence and structural features. Final models are selected by clustering high‐scoring poses, providing representative peptide‐protein complexes [50].

The program can be downloaded from http://wallnerlab.org/InterPep2, allowing users to run it locally on their own systems [50].

A notable strength is that combining InterPep2's template‐based predictions with ab initio predictions from other tools like PIPER‐FlexPepDock can yield even better results. Additionally, its superior ability to evaluate the quality of its predictions sets it apart from other tools. However, a potential limitation is that InterPep2 relies heavily on templates, which might affect its performance for entirely novel interactions with no structural homologs [50].

InterPep2's template‐based approach, combined with machine learning techniques for model evaluation, offers researchers a powerful tool for predicting peptide‐protein interactions. While it has some limitations, its overall performance and flexibility make it an asset for understanding and manipulating protein‐peptide interactions for various biological and therapeutic applications.

### 3.14 PatchMAN

PatchMAN (Patch‐Motif AligNments) is an innovative approach to peptide‐protein docking that takes a unique perspective on the problem. Unlike traditional methods that focus on the peptide's conformation, PatchMAN approaches docking from the viewpoint of the receptor protein. This method, developed at The Hebrew University of Jerusalem and released in 2021, is based on the principle that the protein surface contains sufficient information to determine the peptide's bound conformation of the peptide [51].

PatchMAN models peptide‐protein interactions by identifying structural motifs on the receptor surface and matching them to complementary peptide fragments from known protein structures. The target peptide is then threaded onto these fragments and refined using flexible docking. The final predictions are obtained by ranking and selecting the highest‐scoring models [51, 88].

PatchMAN is a web server that can be accessed at https://furmanlab.cs.huji.ac.il/patchman/, providing a user‐friendly interface for researchers to submit their docking jobs. The receptor must be in a pdb format, while the peptide is inserted as a sequence [51].

PatchMAN's unique approach allows it to sample the bound peptide conformation based solely on the structural context of the receptor without requiring sequence information. However, potential disadvantages might include computational intensity due to its comprehensive surface mapping approach and the maximum receptor length of 500 residues [51].

In conclusion, PatchMAN's receptor‐centric approach offers a novel perspective on the study of these interactions. While it may have some limitations, its overall performance and innovative methodology make it a valuable addition to the toolkit of computational structural biologists and drug designers.

## 4 Integrative Docking Strategies

Peptide–protein interactions are often challenging to model due to peptide flexibility and incomplete knowledge of binding sites. To address these challenges, integrative docking approaches combine multiple computational tools that differ in sampling range and resolution. Global or coarse‐grained methods such as CABS‐Dock, PepATTRACT, HPEPDOCK, or ZDOCK [6, 42, 56, 70], are particularly useful for blind docking, performing extensive searches over the receptor surface to identify potential binding regions and generate diverse starting poses. These methods provide broad sampling but at a lower resolution.

Promising candidate poses can then be refined using high‐resolution local methods, such as HADDOCK, which better account for peptide backbone flexibility, side‐chain conformational optimization, and interface energetics [32]. By focusing computationally expensive refinement on a subset of candidates identified by global searches, this integrative approach combines broad sampling with detailed modeling. Additionally, molecular dynamics simulations are often employed after docking to further refine peptide‐protein complexes, explore conformation flexibility, and assess the stability of predicted interfaces under more realistic conditions [127, 128].

For highly flexible peptides or mobile receptors, ensemble‐based approaches, such as DINC‐ensemble, can incorporate multiple receptor conformations to capture alternative binding modes that single‐structure protocols might miss [67].

Overall, integrative docking strategies leverage the strengths of complementary tools across different sampling ranges and resolutions, from global blind searches to high‐resolution refinements and simulations. By combining these approaches, researchers can improve the reliability and biological relevance of predicted peptide–protein complexes.

## 5 Other Molecular Docking Programs

**FIGURE 3:**
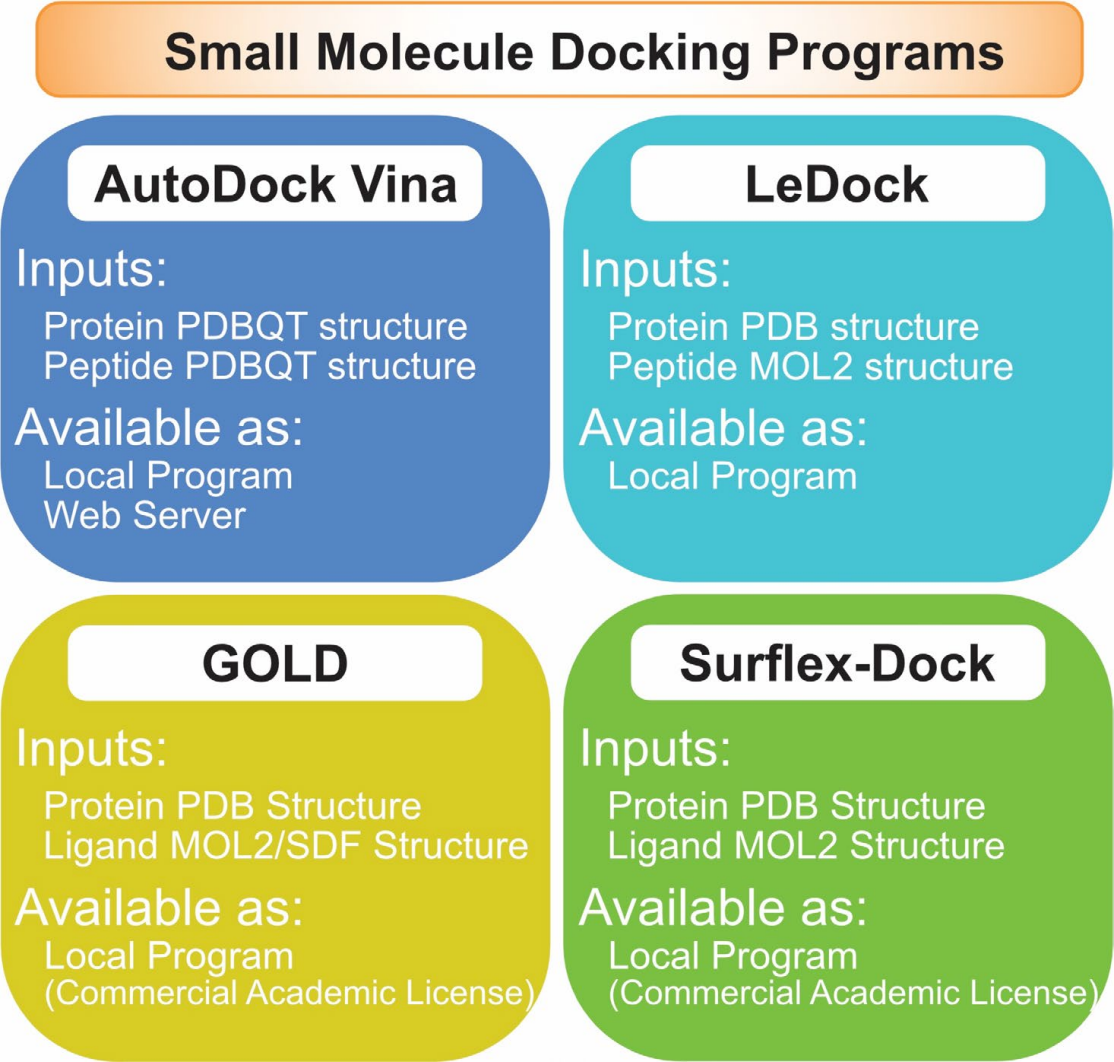
Summary of the small molecule docking programs discussed in this article.

While all the above‐mentioned programs are designed explicitly for peptide‐protein or protein–protein docking, some general‐purpose molecular docking software can also be effectively used for this purpose. These programs, developed initially for small molecule docking, have also been adapted or found to be suitable for peptide‐protein interactions (Figure 3). Here, we will discuss some of these versatile docking programs and their advantages and limitations in the context of peptide‐protein docking.

AutoDock Vina [111] is one of the most popular and widely used docking programs that has shown promise in peptide‐protein docking. In a study which aimed to investigate the inhibitory effects of amino acids and dipeptides on xanthine oxidase and dipeptidyl peptidase IV enzymes, which are relevant to diabetes and oxidative stress management, AutoDock Vina was used to better understand the interactions between the peptides and the active sites of XO and DPP‐IV [129]. Another study investigated the binding interactions of two synthetic antiviral peptides (DET2 and DET4) with the domain III of dengue virus type 2 envelope protein. The researchers used AutoDock Vina to perform molecular docking, generating peptide‐protein complexes and computing their binding affinities. The best‐ranked complexes from the docking simulations were further analyzed using molecular dynamics and MM‐PBSA calculations in order to obtain binding free energies and identify key residues involved in peptide‐protein interactions [130]. Vina employs an efficient optimisation algorithm and an extensively validated scoring function, making it suitable for handling the flexibility of peptides. AutoDock Vina's advantages include its speed, accuracy, and ability to handle larger search spaces, which is particularly useful for peptides with more freedom than small molecules. However, it may struggle with long peptides or those requiring extensive conformational sampling.

GOLD (Genetic Optimisation for Ligand Docking) [9] is another versatile docking program successfully applied to peptide‐protein docking. In a study which focused on designing peptide vaccines targeting breast cancer by predicting their binding to MHC class I and II molecules, GOLD was used to dock a library of 12 peptides. The results provided insights into the specific interactions between the peptides and MHC molecules and revealed one peptide as a good candidate for more analyses [131]. Using computational methods to predict potential Angiotensin‐I converting enzyme inhibitory peptides, researchers constructed an 8000 tripeptide library and used GOLD to study the interactions between ACE and each tripeptide. Based on the *in silico* results, five tripeptides were selected for in vitro ACE inhibition assays, with one having promising results [132]. GOLD's strength is that it includes a variety of scoring functions, allowing users to choose the most appropriate one for their specific case. However, GOLD can be computationally intensive, especially for larger peptides, and may require more extensive parameter optimisation.

LeDock [133] is another docking program with promising results in peptide docking studies. It employs an efficient sampling algorithm and, in a study by Wang et al., has been reported to outperform AutoDock Vina in some cases [134]. However, its performance may decrease for longer peptides or more complex binding sites.

Surflex‐Dock [135], part of the SYBYL suite, has also been used for peptide‐protein docking. In the above‐mentioned study by Wang et al., Surflex‐Dock is the program with the best performance among the studied programs, outperforming Vina, GOLD, and LeDock [134]. A study by Xie et al. focused on enhancing the activity of angiotensin‐I converting enzyme inhibitory peptides through structural modification. The authors designed and synthesized four tripeptides from oyster hydrolysates and used Surflex‐Dock to predict the binding affinities and identify critical interactions between these peptides and ACE [136]. Another study investigated the umami‐enhancing properties of kokumi‐active γ‐glutamyl peptides, focusing on their interaction with the umami receptor T1R3 in the presence of monosodium glutamate. The authors used Surflex‐Dock to simulate binding between various γ‐glutamyl peptides and the T1R3‐MSG complex, identifying critical residues that enhance umami taste [137]. Given that several milk/whey‐derived peptides exhibit high in vitro angiotensin I‐converting enzyme (ACE) inhibitory activity, another study used Surflex‐Dock to investigate whey protein‐derived peptides, predicting their affinity for and interactions with human ACE [138]. Surflex‐Dock's strength lies in its ability to handle ligand flexibility efficiently, which is crucial for peptide docking. However, it may be less effective when significant protein flexibility is involved in the binding process.

While these docking programs offer several advantages, including wide availability, extensive documentation, and often faster computation times, they also have limitations when applied to peptide–protein docking. While the performance of small peptides can be good, one common challenge is the limited ability to handle the extensive conformational flexibility of longer peptides. Additionally, these programs may not account for specific features of peptide–protein interactions, such as the importance of backbone hydrogen bonds or the tendency of peptides to form secondary structures upon binding.

## 6 Artificial Intelligence‐Based Methods

**FIGURE 4:**
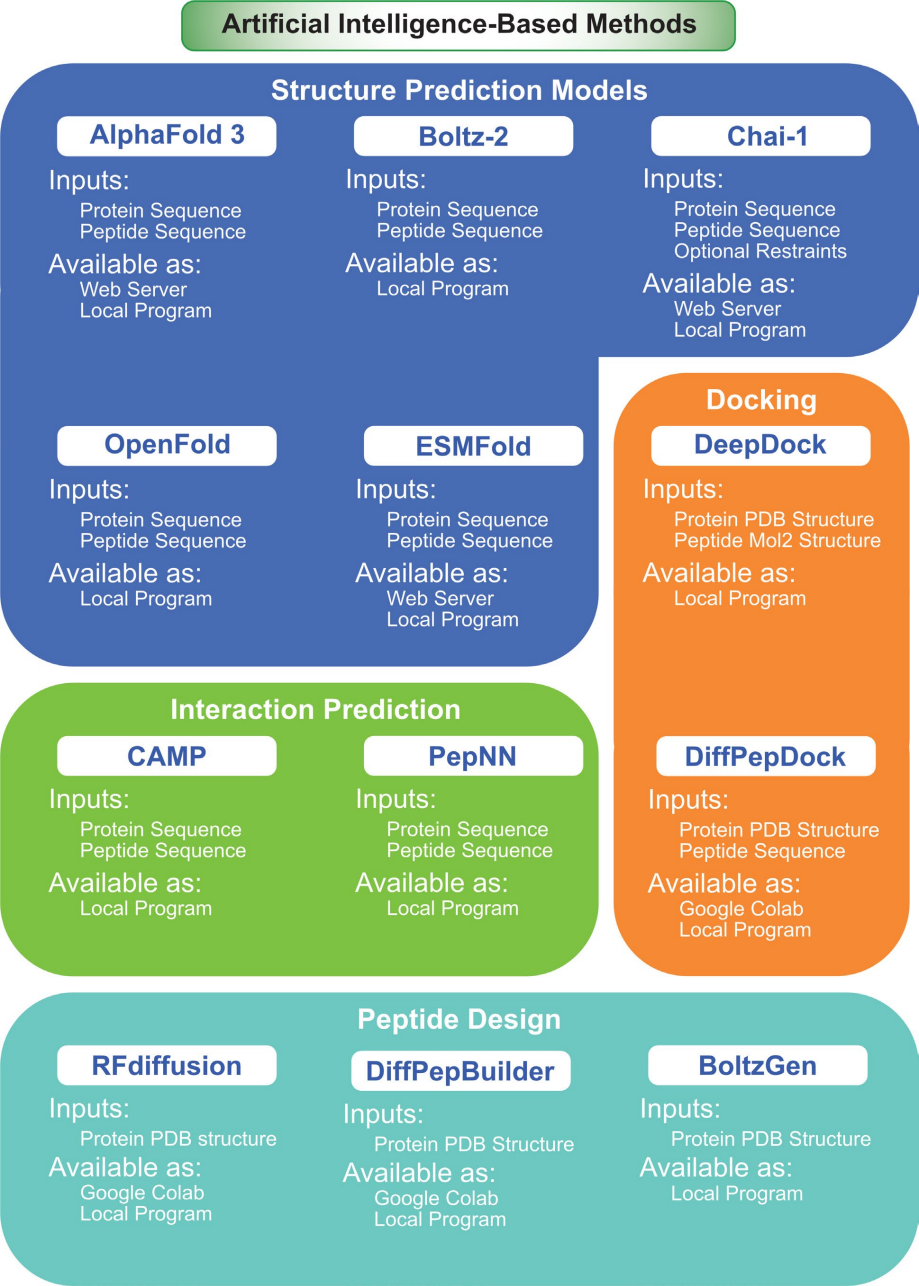
Summary of the Artificial Intelligence (AI)‐based programs discussed in this article.

In recent years, artificial intelligence (AI) methods, particularly machine learning (ML) and deep learning (DL) approaches, have emerged as powerful tools for peptide‐protein docking, offering new possibilities to address the limitations of traditional docking programs. These AI‐driven methods can complement or enhance existing docking protocols, improving accuracy and efficiency in predicting peptide‐protein interactions [139]. In this section, some of these methods will be highlighted (Figure 4).

Alphafold 3 [140] (AF3), released by DeepMind in 2024, represents a major leap forward in protein structure prediction. Building on the foundation laid by AlphaFold 2 [141] (AF2) and AF2 multimer [142], AF3 significantly improves the prediction of complex structures, including peptide–protein complexes. Compared to previous models, AF3 achieves approximately a 10% improvement in accuracy for general protein–protein interactions and up to double the accuracy for specific interactions, all while only requiring the peptide sequence, rather than extensive structural information. This comprehensive approach to modeling diverse biomolecular interactions makes AF3 a powerful tool in computational biology. While AF2 revolutionized the field and earned its developers the 2024 Nobel Prize in Chemistry, AF3 takes this innovation further by expanding predictive capabilities and accuracy.

Following the advances of AF3, new alternatives have emerged. Developed at MIT and released in late 2024, Boltz‐1 became the first widely available, non‐proprietary model to achieve AlphaFold3‐level accuracy in predicting the three‐dimensional structures of proteins, RNA, DNA, and multi‐molecular complexes. The latest iteration, Boltz‐2, raised the bar by not only predicting the three‐dimensional structures but also predicting binding affinity. Boltz‐2 can estimate binding affinities 1000 times faster than other more computationally expensive methods, while its developers claim it is able to maintain strong experimental correlation. Furthermore, being open‐source and releasing both its training data and weights, Boltz‐1 and Boltz‐2 have expanded access to high‐precision biomolecular modeling [143]. Building on the foundation laid by Boltz‐1 and Boltz‐2, BoltzGen is an all‐atom generative diffusion program that can generate new protein binders to any biomolecular target, including proteins, nucleic acids, and small molecules. The first collaborations yielded experimentally validated binders with good affinities, demonstrating the potential of this program. Similar to Boltz‐1 and Boltz‐2, BoltzGen is an open‐source program, available for unrestricted academic and commercial use, supporting broad accessibility and collaborative advancement in protein binder design.

Also released in late 2024, Chai‐1 was developed by Chai Discovery to provide a flexible, multi‐modal platform for biomolecular three‐dimensional structure prediction. Unlike many models that need extensive evolutionary information, Chai‐1 can also be run in single‐sequence mode, making it particularly powerful for predicting structures of orphan proteins or engineered sequences. Additionally, Chai‐1 allows users to incorporate experimental restraints that guide the folding of the complex, further enhancing accuracy in practical applications. Chai‐1 is open source for non‐commercial research and is accessible via an intuitive web interface, lowering the barrier for both academic and industry users to leverage advanced structure prediction in molecular discovery [144]. In June 2025, Chai‐2 was released, claiming unprecedented success rates in the design of *de novo* antibodies. As of the writing of this review, the early access of this new version is available on request for limited non‐commercial use.

The OpenFold project is an open‐source initiative aimed at replicating and extending the capabilities of the AlphaFold models. OpenFold1 focused on creating a trainable and memory‐efficient reproduction of AlphaFold2. Building on this, OpenFold2 improved prediction efficiency and ease of use. With the preview releasing in late 2025, OpenFold3 represents a significant advancement in the field of AI‐driven protein structure prediction. This fully open‐source model allows researchers to customize, retrain, and use this tool on commonly available GPUs. OpenFold3 supports structure prediction of proteins and peptides, but also non‐canonical proteins, RNA, DNA, and small molecules. The OpenFold3 preview performed competitively compared with other similar programs such as AlphaFold3, Boltz‐1, and Chai‐1. OpenFold3 offers a unique combination of flexibility and high prediction accuracy, without the limitations of commercial licensing, standing as a powerful, accessible alternative to proprietary solutions [145].

ESMFold, originally designed for protein structure prediction, can also be used for peptide‐protein docking. Through the use of polyglycine linkers, recycling mechanisms for repeated predictions, and masking strategies to enhance sampling, it has shown promising results. While ESMFold's docking performance is generally lower than that of tools such as AF2 multimer, the computational efficiency of this program makes it potentially suitable for high‐throughput applications [146].

Multiple other AI‐based methods exist, such as CAMP (Convolutional Attention Model for Protein‐Peptide binding), which employs a deep learning framework for multi‐level peptide‐protein interaction prediction. CAMP utilizes convolutional neural networks (CNNs) and self‐attention mechanisms to extract both local and global information from protein and peptide sequences. Furthermore, CAMP can accurately identify peptide‐binding residues using only sequence‐based information as input [147]. DeepDock uses both ligand‐based and structure‐based information as inputs for its neural networks. It then utilizes deep learning models to predict protein‐ligand binding poses [148]. Another AI‐based tool is PepNN. This method predicts peptide‐protein interactions using a Transformer architecture. This architecture primarily consists of repeated multi‐head attention modules. PepNN utilizes both sequence and structural information from protein‐peptide complexes and requires a protein structure and a peptide sequence to perform its prediction [149].

Diffusion‐based programs, such as RFdiffusion [150] and DiffPepBuilder [151], have emerged as powerful tools in peptide‐protein docking by using generative diffusion models to design peptide binders with high specificity and structural accuracy. RFdiffusion generates peptide binders by partially noising and denoising protein structures. This approach has demonstrated success in designing binders with atomic‐level precision, significantly reducing the need for extensive high‐throughput screening [150]. Similarly, DiffPepBuilder employs an SE(3)‐equivariant diffusion model to co‐design peptide sequences for specific protein targets. By incorporating disulfide bonds for enhanced stability, the developers showed that for specific systems, it could outperform methods such as RFdiffusion [151]. In 2025, DiffPepDock, a derivative tool, was launched to perform peptide‐protein docking [152]. DiffPepDock is reported to achieve accuracy comparable to AlphaFold3 predictions but requires less computational time [152]. These diffusion‐based programs represent a significant advancement in computational docking, enabling the efficient design of novel peptides.

These AI methods offer several advantages over traditional docking programs. They can handle the high flexibility of peptides more effectively. AI models can also learn complex patterns and features from large datasets, capturing subtle interaction details that physics‐based scoring functions might miss. Furthermore, once trained, these models can make predictions rapidly, allowing for high‐throughput screening of peptide‐protein interactions. However, AI methods also have some limitations. They heavily rely on the quality and quantity of training data, which can be challenging. There's also the risk of overfitting the training data, potentially limiting the prediction of novel peptide‐protein complexes. Additionally, while AI models can make accurate predictions, they often lack the interpretability of physics‐based methods, making it challenging to understand the underlying principles of the predicted interactions.

## 7 Conclusion

Protein‐peptide docking programs have become essential tools in the field of drug discovery and structural biology. The software packages reviewed in this article each offer unique approaches to addressing the challenges of protein‐peptide interactions. These programs have made significant strides in accommodating the inherent flexibility of peptides and incorporating various types of experimental data. Despite these advancements, challenges remain in accurately predicting protein‐peptide interactions, particularly for highly flexible peptides or in cases with limited experimental data. Future developments in this field will likely focus on improving scoring functions, enhancing the modeling of peptide flexibility, and integrating machine learning approaches to better predict binding modes. As the field of peptide therapeutics continues to grow, these docking tools will play an increasingly crucial role in designing and optimizing peptide‐based drugs. Researchers should carefully consider the strengths and limitations of each program when selecting a tool for their specific needs, as well as testing multiple options in order to find out which better suits the particular study. The ongoing development and refinement of protein‐peptide docking programs will undoubtedly contribute to our understanding of molecular interactions and accelerate the discovery of novel peptide‐based therapeutics.

## Funding

This work was financially supported by national Portuguese funds through FCT/MECI (Fundação para a Ciência e Tecnologia and Ministério da Educação, Ciência e Inovação) under the projects UID/50006/2025—Laboratório Associado para a Química Verde—Tecnologias e Processos Limpos and 2021.07128.BD. Additional financial support was provided by the UMCG Research Funds.

## Conflicts of Interest

The authors declare no conflicts of interest.

## Data Availability

Data sharing not applicable to this article as no datasets were generated or analyzed during the current study.
